# Evaluation of Sex-Aware PrediXcan Models for Predicting Gene Expression

**Published:** 2022

**Authors:** Emily Mahoney, Vaibhav Janve, Timothy J. Hohman, Logan Dumitrescu

**Affiliations:** 1 Vanderbilt Memory and Alzheimer’s Center, Vanderbilt University Medical Center, Nashville, TN 37212, USA; 2 Vanderbilt Genetics Institute, Department of Medicine, Vanderbilt University Medical Center, Nashville, TN 37212, USA

**Keywords:** gene expression, sex differences, PrediXcan, prediction accuracy

## Abstract

Gene-based methods such as PrediXcan use expression quantitative trait loci to build tissue-specific gene expression models when only genetic data is available. There are known sex differences in tissue-specific gene expression and in the genetic architecture of gene expression, but such differences have not been incorporated into predicted gene expression models to date. We built sex-aware PrediXcan models using whole blood transcriptomic data from the Genotype-Tissue Expression (GTEx) project (195 females and 371 males) and evaluated their performance in an independent dataset. Specifically, PrediXcan models were built following the method described in Gamazon et al. 2015, but we included both whole-sample and sex-specific models. Validation was evaluated leveraging lymphoblast RNA sequencing data from the EUR cohort of the 1000 Genomes Project (178 females and 171 males). Correlations (R^2^) between observed and predicted expression were evaluated in 5,283 autosomal genes to determine performance of models. In sum, we successfully predicted 1,149 genes in males and 623 in females, while 3,511 genes appeared to be not sex-specific. Of the sex-specific genes, 15% (189 genes in males and 73 genes in females) exhibited higher R^2^ in sex-specific models compared to whole-sample models, although the overall gain in predictive power was generally minimal and well within measurement error. Nevertheless, two female-specific genes and six male-specific genes showed significantly better prediction when using the sex-specific weights versus the whole-sample weights; furthermore, several of these genes play a role in mitochondrial metabolism, which is known to be influenced by sex hormones. Taken together, these results support previous reports of the small contribution of genetic architecture to sex-specific expression. Still, sex-aware PrediXcan models were able to provide robust sex-specific prediction signals. Future studies exploring the contribution of the X chromosome and tissue specificity on sex-specific genetically regulated expression will clarify the utility of this method.

## 1. Introduction

Sex differences exist in the susceptibility to and progression of many diseases, yet biological sex is typically modeled as a nuisance variable in genetic analyses and rarely integrated into theoretical and analytical models. In fact, embracing these sex differences provides an opportunity to further our understanding of the molecular basis of disease as sex-aware genomic models can increase statistical power by reducing heterogeneity and may more accurately reflect the underlying genetic architecture of sex-biased diseases. Additionally, research assessing factors that influence sex differences in disease, including genetic variation, is necessary for uncovering new therapeutic targets and facilitating precision medicine.

Tissue-specific sex-biased gene expression may contribute to observed sex differences in disease. A recent study, which provided the most extensive characterization of sex differences in the human transcriptome to date, determined that 37% of all genes display sex-biased expression in at least one tissue.^1^ These effects are small and largely tissue-specific. Additionally, the evidence for sex-specific genetic regulation of expression was much more limited. However, the investigators identified 58 gene-trait associations driven by sex-specific gene regulation. These results suggest that while sex differences in gene expression are ubiquitous, sex-specific regulation of gene expression may be more limited, though critical to understand sex-specific genetic contribution to disease.

PrediXcan^2^ is an innovative imputed gene expression technique that can leverage publicly available genotype-linked RNA expression databases like the Genotype-Tissue Expression (GTEx) database.^3^ PrediXcan aggregates the quantitative trait loci (eQTL) from multiple variants into a single functional unit – a tissue-specific estimate of genetically regulated gene expression. This method overcomes the classic pitfalls of genome-wide association study (GWAS) approaches, like implication of non-coding variants of unknown consequences, by placing focus on gene function while also increasing statistical power and reducing the total number of comparisons. PrediXcan has been successfully utilized to interrogate gene-disease trait associations;^4,5^ however, the current standard PrediXcan models do not explicitly model sex-specific effects, particularly among sex-biased genes.

Here, we examine the performance of PrediXcan models applied in a sex-aware manner to evaluate its usefulness and potential for interrogating the sex-specific genetic etiology of disease. We calculate sex-specific and sex-agnostic PrediXcan models in whole blood leveraging data from GTEx focusing on the autosomes. We then validated models leveraging genome-wide genotype data and whole blood RNA sequencing data from the 1000 Genomes Project. We had three goals in our analyses. First, we assessed whether sex-specific PrediXcan models robustly predict gene expression in the appropriate sex in an independent dataset. Second, we assess the prevalence of sex-specific effects across the genome to provide a recommendation about the potential utility of sex-specific PrediXcan models. Finally, we assessed whether there are certain genes that perform better in sex-specific models compared to models that include both sexes to highlight any genes that may benefit from sex-specific prediction.

## 2. Methods

### 2.1. Genomic and transcriptomic data

#### 2.1.1. GTEx

The GTEx dataset was used to build sex-specific weights for gene expression. The data used for analyses were obtained from dbGaP accession number phs000424.GTEx.v8.p2 on 11/13/2019. Genotypes and whole blood RNA sequencing data were prepared following the same pipeline as the original PrediXcan paper reported.^2^ Raw genotypes with minor allele frequency (MAF)<0.01 were excluded, indels were removed and all variants were lifted to genome build 37 to be on the same build as the replication dataset. Samples were restricted to European individuals. Genotypes were imputed to the HRC panel using the Michigan Imputation Server.^6^ Following imputation, variants were restricted to bi-allelic SNPs and limited to those with an imputation R^2^>0.8 and MAF>0.01, leaving 7,760,575 variants and 706 samples to carry forward into model building.

Whole blood RNA expression values were normalized, following the GTEx consortium’s pipeline (https://github.com/broadinstitute/gtex-pipeline/blob/master/TOPMed_RNAseq_pipeline.md). Briefly, across samples, values were normalized to the average empirical distribution and then, within each gene, values were inverse quantile normalized. Genes expressed in at least 10 samples at greater than 0.1 RPKM and greater than 5 reads were carried forward. Then, expression values were corrected using a residual method for 60 PEER factors, sex, genetic principal components, and sequencing platform. In sum, expression for 26,946 genes in 567 samples was leveraged for building models of predicted expression.

#### 2.1.2. 1000 Genomes Project

Genotypes and RNA expression from lymphoblast cell lines (LCL) from the 1000 Genomes Geuvadis dataset were leveraged to evaluate the predictive accuracy of sex-specific models.^7^ Raw genotypes were excluded if missingness>0.05, MAF<0.01, or HWE p<1e-6 and were uploaded for imputation to the HRC reference panel on the Michigan Imputation Server.^6^ After imputation, variants were restricted to bi-allelic SNPs, and variants with an imputation R^2^<0.8 and MAF<0.01 were removed, leaving 7,807,606 variants.

RNA expression values, already QC’ed and normalized, were downloaded from the EMBL-EBI database (https://www.ebi.ac.uk/arrayexpress/experiments/E-GEUV-3/). Out of the 465 samples, we restricted to European individuals with LCL RNA expression data, leaving 358 people (178 females and 171 males) for validation of sex-specific weights from with GTEx data.

### 2.2. Sex-specific estimation of the genetic component of gene expression levels in whole blood

Using the version 8 GTEx RNA sequencing data and genotypes, we rebuilt models of genetically regulated expression in the whole sample as well as in each sex separately, using the original PrediXcan method.^2^ Briefly, this involved running nested cross-validated Elastic Net models. Expression and genotypes were limited to females and to males before running the Elastic Net model to select which SNPs best explained expression within each sex. Models were then filtered using the original thresholds: R^2^>0.01 and p-value<0.05. Source code for the sex-specific model building can be found at the project repository on GitHub (https://github.com/VUMC-VMAC/build_sex_stratified_PrediXcan_weights).

### 2.3. Evaluating performance of sex-specific gene expression prediction

These PrediXcan models for whole blood expression were applied in the 1000G EUR population, giving predicted expression based on weights from the whole sample as well as from males and females separately. Genes with greater than 90% zero predicted expression were removed, as having no informative prediction. We regressed sex-specific and whole-sample predicted expression against actual expression. Then, we compared R^2^ between these models to assess whether the sex-specific predicted expression was more strongly associated with actual expression within the relevant sex than the whole-sample predicted expression. Change in R^2^ was bootstrapped to determine whether any observed improvement was significant. Validated sex-specific models were those where 1) predicted expression positively associated with actual expression (ie, p<0.05 and beta>0) and 2) sex-specific R^2^>whole-sample R^2^.

## 3. Results

### 3.1. Evaluation of sex-specific model performance in GTEx

**Fig. 1.**
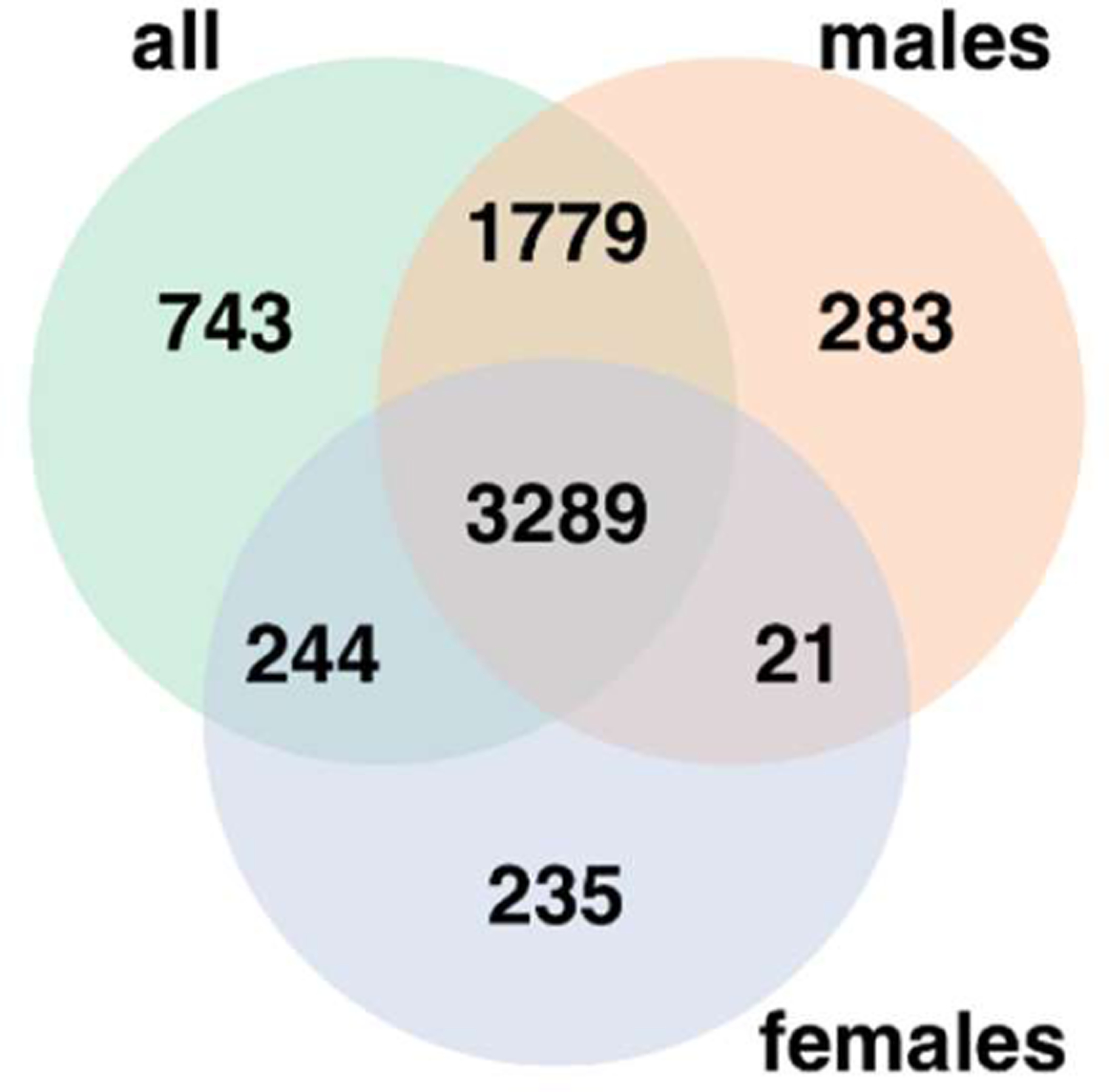
Overlap of Successfully Modeled Genes in GTEx. The number of genes whose gene expression was successfully modeled in GTEx (ie, R^2^>0.01 and p-value<0.05) is presented for the in whole sample (green), males (orange), and females (blue).

Using transcriptomic and genetic data, sex-aware prediction models were built in the GTEx whole blood cohort (195 females, 371 males). The number of successfully modeled genes and model fit metrics (e.g. R^2^) were extracted to assess model performance. Results are presented in Table 1 and Figure 1. Out of 26,946 genes, a total of 6,055 genes were successfully modeled using the standard PrediXcan methodology in all samples regardless of sex, with an average R^2^=0.144. Interestingly, while a slightly smaller number of genes were modeled in the male sample set (5,372 genes), the average model fit was slightly smaller (average R^2^=0.137). In contrast, fewer prediction models (3,789 genes) were built in females compared to the whole-sample set, albeit with a similar average R^2^=0.133. The lower number of gene models built in females compared to males is not unexpected given the smaller sample size in females, thus highlighting the need for larger, more diverse genotype-linked RNA expression databases. Notably, 256 female-specific genes and 304 male-specific genes did not have successful whole-sample models (Figure 1).

**Fig. 2.**
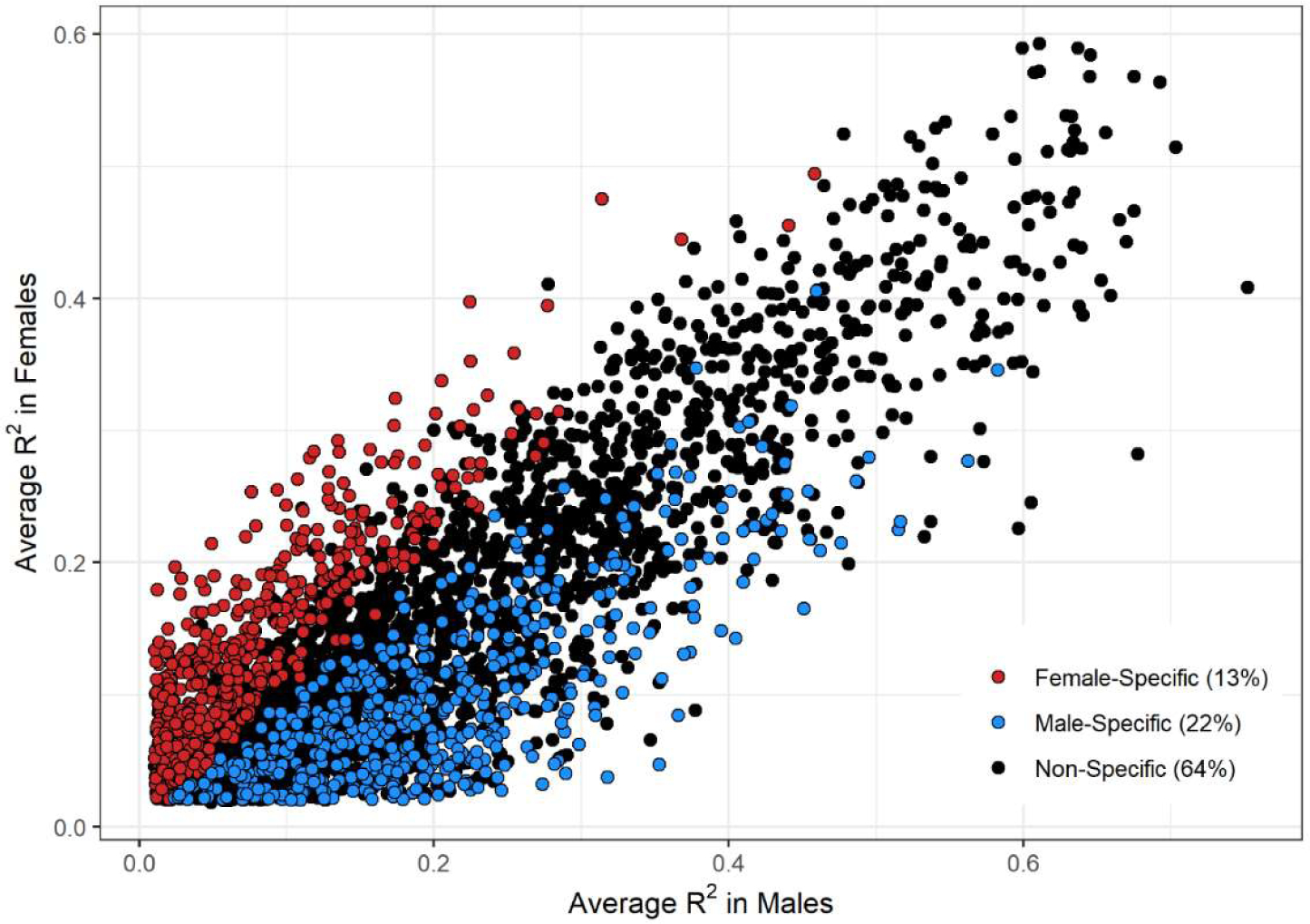
Comparison of Sex-Specific vs Whole-Sample Models. Model fit (R^2^) for each gene in males is presented on the x-axis and for females on the y-axis. Points are colored based on whether they had a better R^2^ from the sex-specific Elastic Net model than from the whole-sample model.

**Fig. 3.**
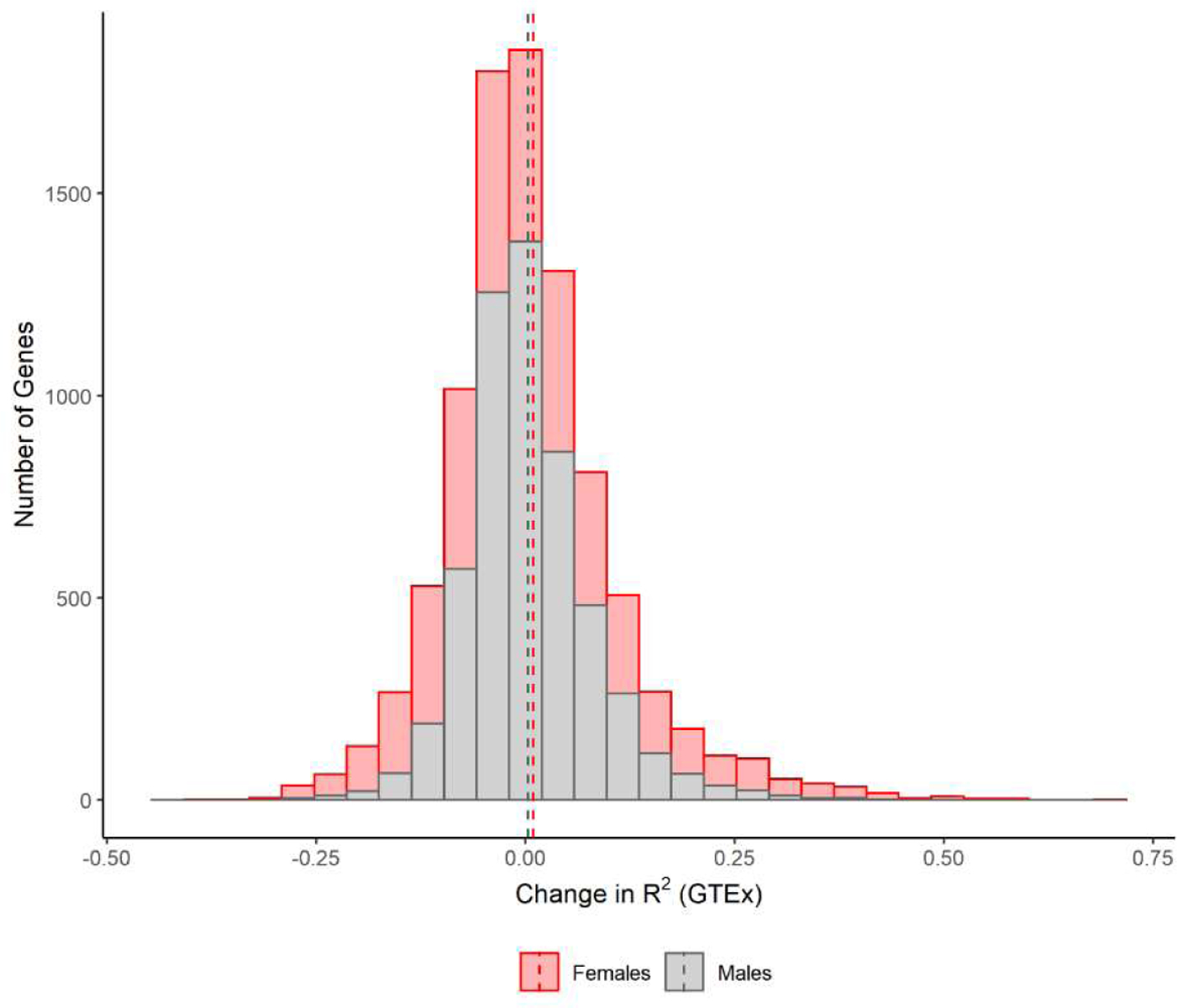
Change in R^2^ from Sex-Specific vs Whole-Sample Models in GTEx. Change in R^2^ (sex-specific R^2^ minus whole-sample R^2^) is presented on the x-axis with the number of genes on the y-axis. Bars are stacked with change in males in grey and change in females in red. Colored vertical lines indicate the average R^2^ change in each sex (0.010 for females, 0.003 for males).

Figure 2 directly compares model fit for each gene model built across the three sample sets. In total, 2,352 (36%) of the modeled genes showed better model fit (i.e., greater R2) in sex-specific builds compared to those built in the whole-sample set. Most of these genes (1,483; 22% of the total) were generated in the male-specific subset, with a smaller proportion in females (869; 13% of the total). It is important to note that, on the individual gene level, the change in R^2^ for the sex-specific models was generally minimal (Figure 3), with only 8% of genes in females and 1% of genes in males having a change in R^2^>0.2. Furthermore, for nearly two-third of the modeled genes (64%), the sex-specific models had equivalent or poorer fit than the whole-sample model.

### 3.2. Prediction accuracy of sex-specific models in 1000G

Using genotype data from the EUR cohort of 1000 Genomes, the sex-aware PrediXcan GTEx tissue models were able to predict expression of 1,772 genes out of the 2,352 sex-specific genes identified in model building, 623 female-specific and 1,149 male-specific. First, we assessed the performance of sex-specific models by directly comparing observed to predicted expression in males and females separately. Overall, sex-aware prediction accuracy was low, with the average R^2^ of 0.03 in both male and female models; albeit 66% of genes did show consistent directions of effect (i.e., positive β) between predicted and observed expression in the male- and female-specific subsets.

**Fig. 4.**
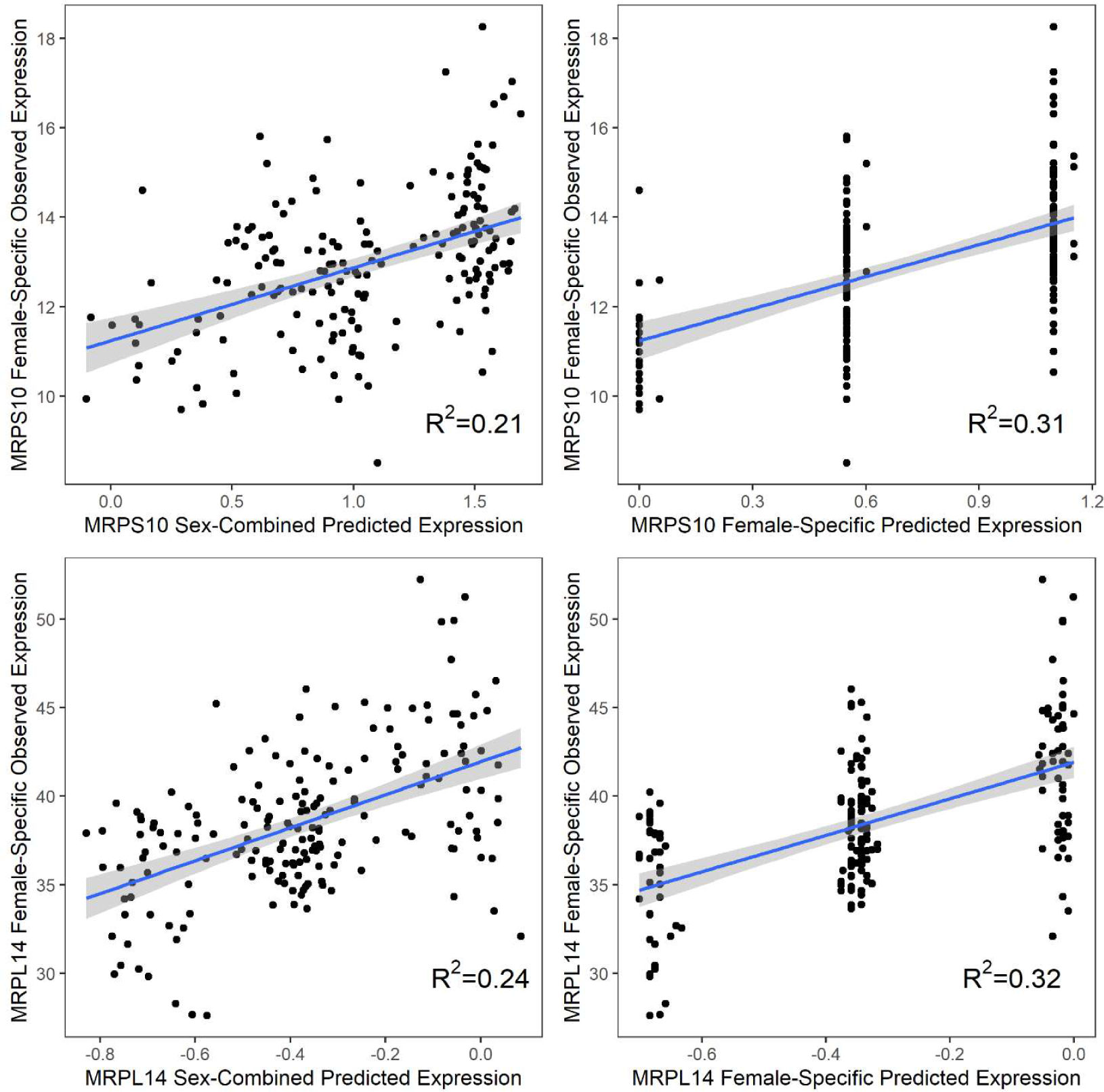
Genes that Showed Significantly Better Prediction Using Female-Specific Models. This figure presents the two female-specific genes which were validated in 1000G, with *MRPS10* in the top row and *MRPL14* in the bottom row. The y-axis in all four plots is observed expression of each gene within females. In the plots on the left, the x-axis is the whole-sample predicted expression (that is, predicted expression based on Elastic Net models with both males and females) while the x-axis in the plots on the right is female-specific predicted expression. The R^2^ for each model is indicated on the bottom right corner of each plot.

Next, we evaluated if we gained any predictive power using the sex-specific PrediXcan models compared to whole-sample models. For most of the genes identified as sex-specific in model building (85%), the sex-specific weights did not perform better than whole-sample weights. Only 15% of the sex-specific gene models were validated in 1000G (73 in females, 188 in males, Table 1), where the predicted expression from sex-specific weights positively associated with actual expression and had a better R^2^ within sex using the sex-specific weights than the whole-sample R^2^. In contrast, of genes with successful whole-sample models, 2,081 genes (41%) replicated in the 1000G dataset, indicating better performance in the whole-sample models. Nevertheless, for the 15% of sex-specific genes which replicated, change in R^2^ was bootstrapped to evaluate whether the sex-specific weights provided significantly better prediction above the whole-sample weights. Two female-specific models and six male-specific models showed significantly stronger associations between actual expression and sex-specific predicted expression than with whole-sample predicted expression (Table 2). Figure 4 shows plots of the two genes, *MRPS10* and *MRPL14*, with significantly better prediction using female-specific models.

## 4. Discussion

We evaluated the reproducibility and value of sex-specific PrediXcan models in two independent datasets. We found that model performance of sex-specific models was largely comparable to the reported reproducibility across sex, suggesting that it is possible to calculate models when only a single sex is available for model building. However, when assessing the prevalence of sex-specific effects and the added predictive value we observed very few genes that showed better prediction in one sex than in the overall sample, consistent with previous reports suggesting genetic regulation of expression is largely conserved across sex. Finally, we highlighted a few autosomal genes that do show sex-specific patterns and may be worth evaluating in a sex-specific manner.

### 4.1. Sex-Specific Models are Reproducible

Overall, models that were successfully estimated in males and females reproduced at a rate that was comparable to previous reports across sex, with the rate of replication increasing with the estimated R^2^ in GTEx. As expected, the number of genes successfully predicted in males and females was a bit lower than in whole-sample models, likely due to the reduction in sample size. In theory, such stratified models should provide increased power in the case of truly sex-specific genetically regulated effects. However, when we assessed the genes that were successfully predicted in one sex but not in the whole-sample models, we did not observe a high rate of replication, suggesting many of these sex-specific models may have been spurious. Perhaps at larger samples it will be possible to build robust sex-specific predictions in some genes that are not predicted across sex, but we do not provide evidence of such sex-specific effects in whole blood. Together, our results suggest that it is possible to build robust predictions in sex-specific models, but that those models do not add a great deal of reproducible genes to the prediction set, at least when restricting model building to whole blood.

### 4.2. Prevalence of Improved Prediction in Sex-Specific Models

Despite limited reports of sex-specific eQTLs,^1^ we did note that approximately one-third of genes showed slightly better prediction in sex-specific models compared to whole-sample models. However, for the vast majority of these genes, the difference in R^2^ was well within measurement error, suggesting there was no true gain in signal. Indeed, out of the 1,772 genes with sex-specific models, only 261 replicated, and only 8 of those genes were statistically significant after bootstrapping. In contrast, for the whole-sample models across sex 41% of signals replicated and 16% of the genes that originally showed stronger prediction in sex-specific models in GTEx were actually more accurately predicted in the whole-sample models. Together, these results suggest that sex-specific models that outperform whole-sample models are largely spurious and do not reflect true biological signal that will reproduce, suggesting whole-sample models should be leveraged for the vast majority of genes in the autosomes.

### 4.3. Exploring the Robust Sex-Specific Prediction Signals Identified in this Study

While most of the sex-specific models appeared to be out-performed by whole-sample models or added very little explanatory power compared to whole-sample models, we did observe a few genes with strong sex-specific genetic effects that may benefit from sex-specific prediction and evaluation when applying PrediXcan in whole blood. It is notable that both genes that showed female-specific genetic regulation (*MRPL14* and *MRPS10*) are members of the mitochondrial ribosomal protein gene family. Mitochondria are critical for energy metabolism and hormone synthesis, and for that reason have been highlighted as critical in understanding sexual dimorphism in disease.^8^ *MRPL14* has not been specifically implicated in sex differences work previously; however, *MRPS10* has been reported to show differential regulation across sexes in colon cancer.^9^ Interestingly, in a Drosophila model of longevity, *MRPS10* showed sex differences in expression and was significantly associated with lifespan determination,^10^ a trait with substantial evidence of sexual dimorphism across species and across human populations. In addition to the MRP genes, we observed a significant gene that encodes a mitochondrial pyruvate carrier in males (*MPC2*), along with multiple genes involved in amino acid and nucleotide metabolism (e.g., *BCKDHA*, *LHPP*, and *HACD3*) that are relevant to the bioenergetics of the cell. Our results highlight genes that are involved in mitochondrial metabolism as novel candidates for sex-specific genetic regulatory effects that may be relevant to disease. Independent replication and fine-mapping approaches, especially for sparse prediction models based on a small number of eQTLs, will be required to better understand the context and causes of improved sex-specific performance for the few genes identified here.

### 4.4. Strengths and Weaknesses

While we did not observe robust replication of sex-specific predicted expression in the current study, there are a few key limitations which could explain this. Specifically, in the current analyses, we were not able to build models of genetically regulated expression for the X chromosome and, since this is where the majority of sex-differentially expressed genes are located,^1^ it is likely that this would also be where the largest impact of genetic regulation would be. Further, sex-differential expression is largely tissue specific, hence these results are only applicable to the tissues studied here. Depending on the phenotype or disease under consideration, the sex differences in regulation in other tissues, like brain for neurodegenerative diseases, breast tissue for breast cancer, or lung tissue for respiratory disease, would be more particularly relevant, though validation datasets for these tissues are difficult to find. Additionally, the samples analyzed are from participants of European descent, thus limiting the applicability of these models to other racial/ethnic groups. Another limitation in this study was sample size. Especially because the effect of genetic regulation is often a relatively small impact on the total variation, larger sample sizes for building models of genetically regulated expression would better capture true signals. Furthermore, differences in sample sizes between the whole-sample and the sex-specific sample sets could contribute to the differences in the prediction performance we observed. Unfortunately, the question of whether the loss in power (due to reduced sample size following sex stratification) is worth the gain in predictive performance will always be an issue for sex-aware analyses.

Our choice of eQTL database for validation has both benefits and limitations. Validation was based on gene expression in LCL, while PrediXcan models were built in whole blood. We chose the LCL dataset because lymphoblasts are the most similar tissue type to whole blood and the dataset is relatively large when compared to other datasets. Nevertheless, tissue-specific differences in gene expression may have contributed to the minimal validation observed in the whole-sample dataset. That being said, the main motivation of this study was not to validate prediction models across tissue types but to compare sex-specific model performance to whole-sample model performance in the same tissue. Thus, differences in gene expression across tissues are less of a concern in our study as they are expected to present in both the whole-sample and sex-specific datasets. To test this assumption, we applied sex-specific prediction models in a small cohort of older adults (99 males and 71 females) with whole blood RNA sequence data. We saw a similar pattern of limited validation in sex-specific models, with 36% of sex-specific predicted models showing evidence of association between observed and predicted gene expression (data not shown), slightly larger than the 15% in the present analysis in LCL. We are currently working to generate and access additional whole blood datasets to validate our findings in the same tissue.

In this study we used Elastic Net to build our whole-sample and sex-specific gene expression prediction models. The Elastic Net algorithm is commonly used for transcriptome imputation due to its prediction accuracy and its robustness to the sparse local architecture of gene expression traits. However, as recently discussed by Barbeira et al,^11^ there are now multiple model training schemes available, some of which have been shown to improve prediction performance and detection of gene-trait association. For example, there are strategies that leverage multi-tissue gene expression (UTMOST^12^), integrate atlases of regulatory elements (JIT^13^), or utilize an entirely different prediction algorithm based on multivariate adaptive shrinkage (*mashr*^14^). Our sex-aware model building approach could also be applied to these different strategies as an important future direction.

### 4.5. Conclusion

This study is the first to explore the potential of sex-specific PrediXcan builds. While we observed evidence of sex-specificity in the genetic architecture of gene expression for a limited number of genes, this study does not provide evidence supporting the wide-spread application of sex-specific PrediXcan builds in the interrogation of genetic drivers of sex-biased diseases. Future work will require integration of the X-chromosome, exploration of more disease-relevant tissues, and testing of models in larger eQTL databases as they become available.

## Figures and Tables

**Table 1.** PrediXcan Model Characteristics and Performance

Group	N Samples	N Genes	Mean R^2^ (SE)	Range R^2^ (min-max)

Model Characteristics in GTEx*				

all	566	6055	0.144 (0.002)	0.010 – 0.768
males	371	5372	0.137 (0.002)	0.010 – 0.752
females	195	3789	0.133 (0.002)	0.019 – 0.593

Models Validated in 1000G				

all^†^	349	2081	0.095 (0.003)	0.011 – 0.852
males^††^	171	188	0.091 (0.007)	0.023 – 0.620
females^††^	178	73	0.072 (0.009)	0.021 – 0.357

* Limited to models with R^2^>0.01 and p-value<0.05

† Limited to validated models, where 1) predicted expression positively associated (ie, p<0.05 and beta>0) with actual expression and 2) whole-sample R^2^>sex-specific R^2^

†† Limited to validated models, where 1) predicted expression positively associated (ie, p<0.05 and beta>0) with actual expression and 2) sex-specific R^2^>whole-sample R^2^

**Table 2.** Sex-Specific Genes

	Whole Sample			Males			
	N SNPs	GTEx R^2^	1000G R^2^	N SNPs	GTEx R^2^	1000G R^2^	R^2^ Change

*HACD3*	12	0.055	0.163	9	0.080	0.184	0.021 [0.001, 0.040]
*LHPP*	40	0.210	0.076	34	0.219	0.102	0.025 [0.001, 0.047]
*MPC2*	7	0.016	0.021	24	0.042	0.096	0.048 [0.001, 0.094]
*DOK6*	51	0.132	0.115	32	0.179	0.142	0.060 [0.015, 0.098]
*BCKDHA*	46	0.151	0.016	18	0.155	0.051	0.038 [0.002, 0.070]
*lnc-GLRX5–6*	14	0.276	0.104	11	0.282	0.142	0.027 [0.004, 0.047]

	Whole Sample			Males			
	N SNPs	GTEx R^2^	1000G R^2^	N SNPs	GTEx R^2^	1000G R^2^	R^2^ Change

*MRPS10*	67	0.282	0.213	51	0.313	0.306	0.089 [0.048, 0.129]
*MRPL14*	40	0.065	0.241	28	0.163	0.322	0.054 [0.014, 0.097]
